# Massively parallel quantification of phenotypic heterogeneity in single-cell drug responses

**DOI:** 10.1126/sciadv.abf9840

**Published:** 2021-09-17

**Authors:** Benjamin B. Yellen, Jon S. Zawistowski, Eric A. Czech, Caleb I. Sanford, Elliott D. SoRelle, Micah A. Luftig, Zachary G. Forbes, Kris C. Wood, Jeff Hammerbacher

**Affiliations:** 1 Department of Mechanical Engineering and Materials Science, Duke University, Durham, NC 27708, USA.; 2 Celldom Inc., San Carlos, CA 94070, USA.; 3 Department of Microbiology and Immunology, Medical University of South Carolina, Charleston, SC 29425, USA.; 4 Department of Molecular Genetics and Microbiology, Center for Virology, Duke University, Durham, NC 27708, USA.; 5 Department of Pharmacology and Cancer Biology, Duke University, Durham, NC 27708, USA.

## Abstract

Single-cell analysis tools have made substantial advances in characterizing genomic heterogeneity; however, tools for measuring phenotypic heterogeneity have lagged due to the increased difficulty of handling live biology. Here, we report a single-cell phenotyping tool capable of measuring image-based clonal properties at scales approaching 100,000 clones per experiment. These advances are achieved by exploiting a previously unidentified flow regime in ladder microfluidic networks that, under appropriate conditions, yield a mathematically perfect cell trap. Machine learning and computer vision tools are used to control the imaging hardware and analyze the cellular phenotypic parameters within these images. Using this platform, we quantified the responses of tens of thousands of single cell–derived acute myeloid leukemia (AML) clones to targeted therapy, identifying rare resistance and morphological phenotypes at frequencies down to 0.05%. This approach can be extended to higher-level cellular architectures such as cell pairs and organoids and on-chip live-cell fluorescence assays.

## Teaser

A live-cell analysis platform measures growth rate and phenotypic properties of up to 100,000 clones per experiment.

## INTRODUCTION

In recent years, advances in sequencing technology have enabled deep, clonally resolved views into the genomic and transcriptional heterogeneity that exists within cellular populations (*1*–*4*). This variance is important, as it likely drives much of the phenotypic heterogeneity that underpins physiological and pathological programs (*3*–*5*). While single-cell genomic tools can now routinely measure the mutational or transcriptional profiles of >100,000 individual cells in a single experiment (*6*, *7*), similar tools for measuring single-cell phenotypic heterogeneity and dynamics remain elusive because of the complexities of working with live biology. One promising approach for capturing phenotypic heterogeneity on a massive scale entails organizing a high-density array of individual cells that can be continuously observed over time microscopically with or without chemical or physical perturbations. Imaging these isolated clones can reveal phenotypic distributions, including rare phenotypes of biological significance, such as cells that respond uniquely to stimuli or produce distinct secreted factors (*8*, *9*). However, to date, no existing platforms have demonstrated the ability to measure single-cell phenotypes at throughputs approaching the 100,000 clone scale. The only platform that approaches this benchmark is the Berkeley Lights Beacon instrument, but to our knowledge, that platform is currently unable to perform more than four parallel experiments per instrument, limiting the ability to analyze phenotypic responses of diverse cell types to assorted stimuli (*9*).

In cancer, rare clones that survive in the presence of chemotherapeutic treatments often drive recurrence of drug-resistant disease (*10*, *11*). In the laboratory, these clones have traditionally been isolated and studied individually through a weeks- to months-long process of selection, enrichment, and clonal isolation. As a result, it has been difficult to quantify the abundance of resistant clones in a population, directly define clonal growth properties, or scale analyses to different tumor samples, cell lines, drugs, and doses. Given the complexity and heterogeneity of resistant clones within individual patients, it is expected that new, integrative approaches will be necessary to design drug therapies capable of suppressing the collective growth of resistant subclonal populations. This necessity underscores the importance of technologies that can measure these properties at scale (*10*–*12*).

Here, we present the first single-cell phenotyping platform that can reach the scale of 100,000 clones in a single, multiday, time-resolved experiment, all performed in parallel by one instrument. Our approach is made possible by fundamental advances in microfluidic chip design, improvements in methods for long-term culturing and microscopic observation of single cells, and finally advances in image processing and analysis software that allow large image-based datasets to be automatically analyzed down to the level of individual cell morphology. Specifically, we report on a previously unidentified microfluidic design that represents the most efficient microfluidic trapping architecture to date, and we also demonstrate robust, cost-effective methods for maintaining mammalian cells on-chip over sufficient time to identify rare phenotypic properties like drug resistance. These results pave the way for more efficient methods for credentialing drugs and, ultimately, improved selection of therapeutic regimens for patients. More broadly, by enabling flexible phenotypic single-cell profiling at massive scale, this platform may facilitate the functional characterization of diverse and complex cellular populations.

## RESULTS

### A previously unidentified microfluidic flow regime enables high-efficiency single-cell trapping

The microfluidic weir trap architectures that have previously been used to organize single cells in an ordered array fall into three fundamental classes. Our classification scheme is based on determining the equivalent circuit representations of each fluidic architecture by identifying the branching points in the flow path (i.e., nodes) and representing the fluid flow along the paths connecting the nodes as equivalent resistors. The flow profiles in the resistor network can be solved by standard matrix solvers in many cases analytically.

For example, the trapping geometry used in the Fluidigm C1 instrument, and also studied by others (*13*), can be equivalently represented as a series arrangement of parallel resistors. In each unit cell, the flow splits into two parallel paths, with one flow path sweeping through the weir trap and the other path following the bypass (resistors in parallel). These flows then join at the opposite side before impinging on the next trap (resistors in series). The cell trapping efficiency in these circuits can be improved by lengthening the bypass channel to increase its resistance, causing more fluid to flow through the trap, but at the expense of reduced trap density in the array.

To overcome the issue of low trap density, an alternative architecture was reported by the Lu group and others (*14*–*19*), which can be approximately modeled as many parallel traps distributed along a common path between the inlet and outlet. The equivalent circuit for these types of fluidic circuits has been analyzed by others (*20*), and the result reveals strong inhomogeneity in the flow profiles across the different traps in the array—this is not ideal for cell culture because it leads to variable conditions experienced by the different clones in the array.

**Fig. 1.**
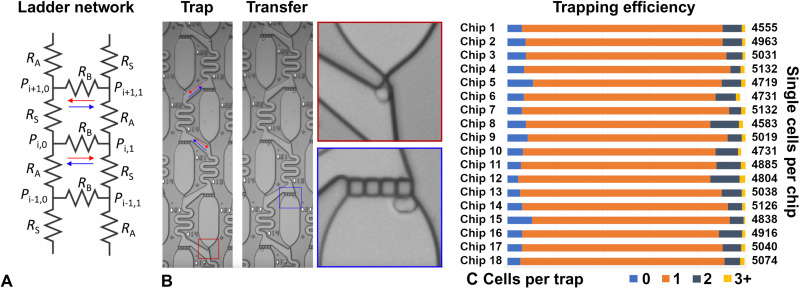
Working principle. The microfluidic device can be modeled as a ladder resistor network (**A**), which has two flow regimes that have high (or low) cell trapping efficiency. The high (low) trapping flow regimes are depicted by the red (blue) arrows, respectively, in which the fluid joins (splits) before entering the apartment. (**B**) The cells are first trapped in the constriction (top) and then transferred into the apartments with a brief pressure pulse (bottom). A higher-magnification view of each step in the trap and transfer process is shown. (**C**) The results from one experiment consisting of 18 chips capturing a total of 88,317 cells show that, on average, 83% of apartments contain a single cell (orange bars), 7% are empty (light blue bars), and 10% have more than one cell [dark blue bars (two cells) and gold bars (more than two cells)]. Scale bar, 100 μm.

In this work, we investigate the third class of weir traps that have a hexagonal arrangement, which leads to more uniform flow profiles across each trap, which we expected to improve performance in long-duration cell cultures. These flow architectures can be arranged in one-dimensional (1D) ladder or 2D mesh resistor networks, and they are unique in that they support two flow regimes depending on the ratio of the resistance in the bypass relative to that of the trap. The transition between the two flow states is achieved by tuning the fluidic resistances in the rails of the ladder (Fig. 1A; see the Supplementary Theory for details), which, in turn, causes the flow direction in the rungs of the ladder to change directions. Unexpectedly, all prior works have universally adopted the less efficient trapping regime (*21*–*25*), in which the resistance through the apartment, *R*_A_ (one of the rails of the ladder or mesh network), is higher than the resistance through the serpentine bypass section, *R*_S_ (the opposite ladder rail). Another way to view the different flow states is that the pressure at the exit of one trap can be higher (or lower) than the pressure at the entrance of the next adjacent trap. If the pressure is higher (lower), then the flow in the short bypass will point toward (away from) the entrance of the adjacent trap, as depicted by the red (blue) arrows in Fig. 1 (A and B). Because the flow joins together at the entrance of each apartment (rather than splitting), it becomes a perfect trap in the mathematical sense because all of the impending flow sweeps through the apartment. This improved trapping efficiency comes at the expense of a larger device footprint, because it is necessary to design longer and narrower channels for the serpentine bypass section to achieve the required resistance ratio. However, this approach is advantageous when the goal is to improve the trap occupancy rate and make more efficient use of limited cell samples.

The working method of this trapping approach is based on a self-limiting principle, in which the fluid flow is modulated by the cell’s physical presence in a trap, which functions as a self-limiting switch to alternate between the two flow states. When the trap is initially empty, it is in a high-efficiency flow state for capturing cells, where *R*_A_ < *R*_S_. After a trap has captured a cell, the flow profile changes because the cell’s physical presence modifies the resistance through the trap, thus switching the flow state to the low-efficiency capture state, where *R*_A_ > *R*_S_. The high resistance of the occupied traps causes subsequent cells to bypass the occupied traps and diverts them downstream toward unoccupied traps. As a result, the cells populate the array in a deterministic fashion, with most of the traps becoming filled in the order that cells were introduced onto the chip.

To load the cells onto the chip, a cell suspension at a concentration of 10^6^ cells per milliliter is prepared in a 0.2 μm–filtered aliquot of cell culture media and then a 10- to 20-μl aliquot of cell suspension is placed into the inlet reservoir, after which it takes approximately 3 to 5 min to fill all of the traps by applying negative pressure (20 to 50 mbar) at the microfluidic outlet. The remaining cells are then rinsed from the device by washing the inlet several times and then flowing clean filtered media through the chip for another minute, leading to a trapping distribution similar to that shown in Fig. 1B (top).

Once the traps are filled, the cells are transferred into the apartments by applying a subsecond elevated pressure pulse, which squeezes the cells through the constrictions into the adjacent apartments. These mechanical perturbations are benign and have been successfully used in various drug and gene delivery applications (*26*–*28*). As a general rule, we found that a 1:3 or 1:4 ratio for the width of the trap region compared to the diameter of the cell was ideal; this allowed cells to be consistently trapped and retained at low pressures (~20 mbar) but reliably transferred into the apartments at higher pressures (~500 mbar). In our chip designs, the single-cell capture efficiency worked best when the front trap width is in the range of 3 to 6 μm, which can be tuned for different cell types having diameters in the range of 10 to 25 μm. Representative images of the cell positions during each step of the trap and transfer process are shown in Fig. 1B.

We also developed automated methods for reading the individual apartment addresses from the images and quantifying the number of cells in each apartment through brightfield image classification techniques. These classifiers are based on standard image segmentation models that have been trained to detect the instances of each cell in each apartment at each time point (*29*, *30*), as described in detail in Materials and Methods. With this software package, we were able to quantify the trapping efficiency in the array and assess any spatial biases that were used to improve the microfluidic architecture.

In this platform, we are simultaneously optimizing two metrics of performance, namely, (i) the number of traps that end up capturing a single cell (typically ~80% in our hands) and (ii) the number of cells needed to completely fill all of the traps, which is related to how the cells are distributed in each of the parallel channels during the loading process. Both of these parameters need to be optimized to effectively make use of limited cell samples. Because this design is very efficient at capturing cells, the 6016 traps in the device are consistently filled when ~10,000 cells are introduced to the inlet. Because of the combination of fabrication defects, presence of debris in the cell culture media, and incompletely dissociated cell suspensions, the trap occupancy for MOLM-13 cells was found, on average, to yield ~80% single cells, ~10% empty apartments, and ~10% apartments having more than one cell (Fig. 1C).

### Cell pairs and reproducible cell clusters can be organized with high efficiency

**Fig. 2.**
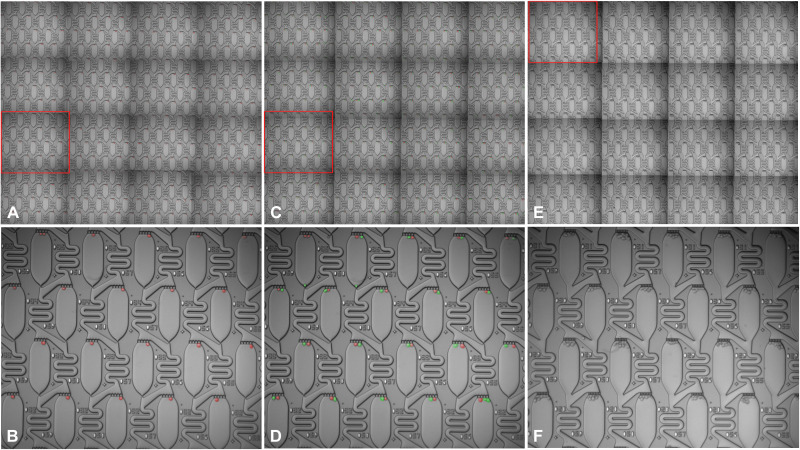
Organization of single cells, cell pairs, and multicellular architectures. After the first loading step in a typical field of view containing ~320 apartments (**A**), the percentage of apartments with empty, singlets, doublets, and multiplets are 2.2, 86.5, 9.8, and 1.6%, with a zoomed-in view of the section highlighted by the red rectangle shown in (**B**). The cells are pseudo-colored red for easier visualization. (**C** and **D**) After the second loading step in the same field of view, the percentage of apartments with a single cell pair (one per each trapping step, i.e., 1:1) was 72.3%, while the next highest percentages for cell pairing formats of 1:2, 2:1, 1:0, 0:0, 3:1, and 1:3 were 9.5, 9.5, 2.9, 2.0, 1.6, and 1.0%, respectively. The cells assembled in the second trapping step are pseudo-colored green. (**E** and **F**) Highly reproducible cell clusters are created in a single shot when the front trap is opened up.

This platform has the ability to organize other types of cellular architectures for myriad potential cellular analysis applications by tuning the device geometry and/or serially repeating the trap and transfer process. The ability to form heterogeneous cell pairs (Fig. 2, A to D), for example, has potential applications in immune oncology and in forming different types of cellular microenvironments. A similar approach has been used by others for fabricating hybridomas (*21*) and for pairing T cells with other cells (*31*). We demonstrate this pairing ability by first organizing an array of MOLM-13 cells (pseudo-colored red) and then repeating this process with the same cells (pseudo-colored green). For a section of the array consisting of ~320 apartments (Fig. 2, A to D), we obtained ~86% single cells and 72% single-cell pairs.

This device can be modified to form reproducible cell groupings in a single shot by opening up the front trap, as shown in Fig. 2 (E and F). For this geometry, repeatable clusters of 6 to 10 cells per apartment were organized reliably across the entire chip, and this approach may have potential applications for rapidly creating spheroids or organoids in a highly parallel format.

### Rare cell phenotypes are observed in multiday cell culture experiments

The chips are fabricated by deep reactive ion etching (DRIE) of silicon wafers, then anodically bonding the wafers to glass lids, followed by dicing the wafers into individual chips, and finally assembling the chips into a custom-machined microfluidic chip holder (see Materials and Methods for detailed fabrication process). This fabrication approach can be readily accomplished in a standard university cleanroom and allowed us to fabricate features as small as 2 μm. The microfluidic architecture was designed such that cells were able to squeeze through the front traps having 3- to 6-μm constrictions; however, cells were retained by the parallel frit structure at the back of the apartments that had smaller 2-μm constrictions. This geometry allowed fluid to pass through the apartments while retaining the cells inside the apartments over many days, enabling the study of clonal growth patterns and variability in morphological features.

To achieve steady perfusion of media into the chips while inside the incubator, we connect 10-ml syringes to the inlet and outlet, which serve as media reservoirs. We fill the inlet syringe with media while the outlet syringe is connected to a vacuum line. In this way, we ensure that the media reservoir at the inlet has unimpeded gas exchange with the ambient conditions inside the incubator. We maintain good cell viability by using weak vacuum pressures in the range of −20 to −50 mbar to continuously flow media through the device. For this specific chip, an optimal flow rate of ~5 ml per day is sufficient to remove metabolic waste products and provide fresh nutrients. However, the exact flow rates need to be tuned for other microfluidic geometries, cell types, and other experimental parameters.

The simplicity of this microfluidic design allows resealable connections to be made easily between the chip and the external pressure controllers, which enables many on-chip experiments to be conducted in parallel (fig. S1A). Each day, the chip is disconnected from the pumping system to perform imaging on a standard fully automated microscope (fig. S1B). To rapidly acquire high-resolution images of each apartment in the chip, we developed imaging algorithms that use microscope image quality focus classifiers (*32*) and image segmentation computer vision models (Mask R-CNN) (*33*) to identify fiducial marks on the chips and determine the optimal focus for each image [see Materials and Methods and GitHub repository posted online (*34*)]. As a compromise between image quality and speed, we opted to perform microscopy at ×10 magnification, allowing the entire chip to be tiled with ~300 images, where 20 apartments are captured in each field of view. This approach allows us to image each chip within 5 to 10 min, depending on the number of fluorescent channels, and it provided the bandwidth to image up to 18 chips per day (see Fig. 1C and fig. S1).

**Fig. 3.**
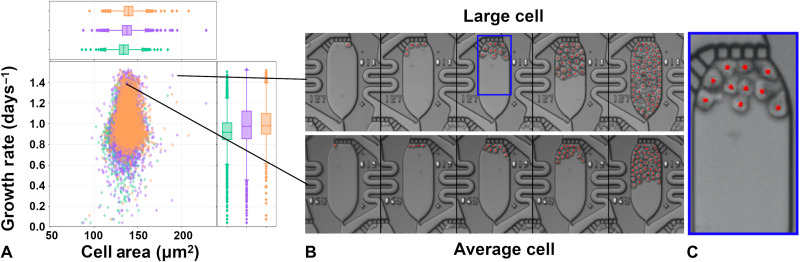
Phenotypic heterogeneity measured in extended duration culture. MOLM-13 cells were grown for 96 hours under continuous perfusion with RPMI 1640 media with 10% fetal bovine serum. The growth rate distribution versus time-averaged cell area across three chips (**A**), with example views of the average cell morphology compared to a rare subset of substantially larger cells (**B**), which were present at frequencies of approximately 0.05%. The time-lapse images are taken at 24-hour intervals, and red dots are added to the images to depict the locations of the cell centroids as identified by the image analysis software. (**C**) Higher-magnification view of the cells with pear-shaped morphology.

With the ability to repeatedly image many chips in parallel over many days and analyze cell properties per apartment with our software pipeline, we have the statistical power to discover rare phenotypic variants of biological significance. For example, in Fig. 3A, we plot the growth rate as a function of the time-averaged mean cell area across the 11,094 single-cell clones that maintained positive growth rates over 96 hours. The distribution of growth rates in each of the three chips shows similar phenotypic distributions, each displaying medians of ~0.95 cell divisions per day, with the middle quartiles falling in the range of 0.84 to 1.12 cell divisions per day and the fastest growth rate exceeding 1.5 cell divisions per day. Because dead cells were removed from the apartments during the continuous microfluidic perfusion conditions, we did not need to train the classifier to distinguish between live and dead cells in the array.

Beyond growth measurements, we found an interesting subset of clonal cellular populations that were not only fast growers but also abnormally large compared to the bulk population. A few of these rare phenotypes were found in each chip, and their frequency in the parental line was assessed to be ~0.05% for this cell line (see Fig. 3B). These rare cells consistently presented with a pear-shaped morphology (Fig. 3C), and the fact that they are larger across all time points and have similar morphologies provides intriguing evidence of (epi)genetically heritable cell size and shape regulation (*35*).

### In situ fluorescence staining extends capabilities of high-throughput single-cell culture

**Fig. 4.**
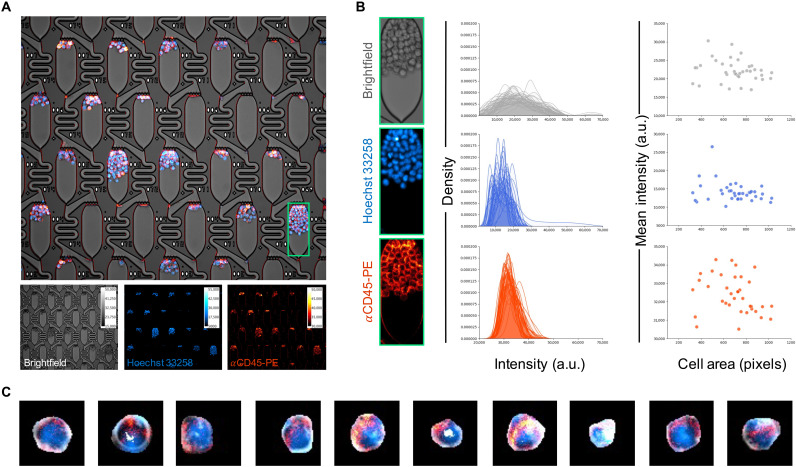
High-throughput extraction of clonal fluorescence data. (**A**) Multichannel live-cell imaging of MOLM-13 clonal populations. After 72 hours of culture under constant flow, cells were stained in situ with Hoechst 33258 to visualize nuclei and PE-conjugated antibody against CD45, a pan-hematopoietic cell surface marker. (**B**) Signal quantification of MOLM-13 cells within a single culture apartment. Density plots depict brightfield, nuclear, and surface marker stain intensity distributions of automatically segmented individual cells within the selected apartment. Scatter plots present the relationship between cell size and mean intensity in each imaging channel. a.u., arbitrary units. (**C**) Example multichannel images demonstrating diversity of individual cells segmented using Mask R-CNN.

In addition to time-resolved studies of clonal growth rates, the microfluidic platform is readily adapted for fluorescence imaging studies, including in situ live-cell staining. In one demonstration, MOLM-13 cells were treated with a cell membrane–permeable nuclear stain (Hoechst 33258) as well as a phycoerythrin (PE)–conjugated antibody against CD45, a marker expressed on all hematopoietic cells (Fig. 4A). Paired with Mask R-CNN cell instance segmentation, this experimental design can illuminate aspects of individual cell morphology and biomarker expression at high throughput on a clonal basis, including clonal phenotypic diversity. As an example, individual cell stain intensity distributions and signal statistics can be extracted for apartments of interest to study quantitative phenotypic differences within or across clones (Fig. 4, B and C).

### Rare drug-resistant phenotypes are observed in multiday cell culture experiments

The power of this platform to analyze thousands of cells per chip and many chips per system makes it uniquely suited for drug screening applications that require single-cell resolution. To demonstrate this approach, we conducted an eight-chip study of cells exposed to either dimethyl sulfoxide (DMSO) or 0.5 or 1.5 nM of the FLT3 inhibitor quizartinib (AC220). MOLM-13 cells harbor the internal tandem duplication (ITD) in-frame insertion in *FLT3*, a gene mutated in ~30% of acute myeloid leukemia (AML) patients and associated with poor prognosis (*36*). ITD renders FLT3 hyperactive via ligand-independent phosphorylation; thus, MOLM-13 cells are exquisitely sensitive to quizartinib (*37*).

**Fig. 5.**
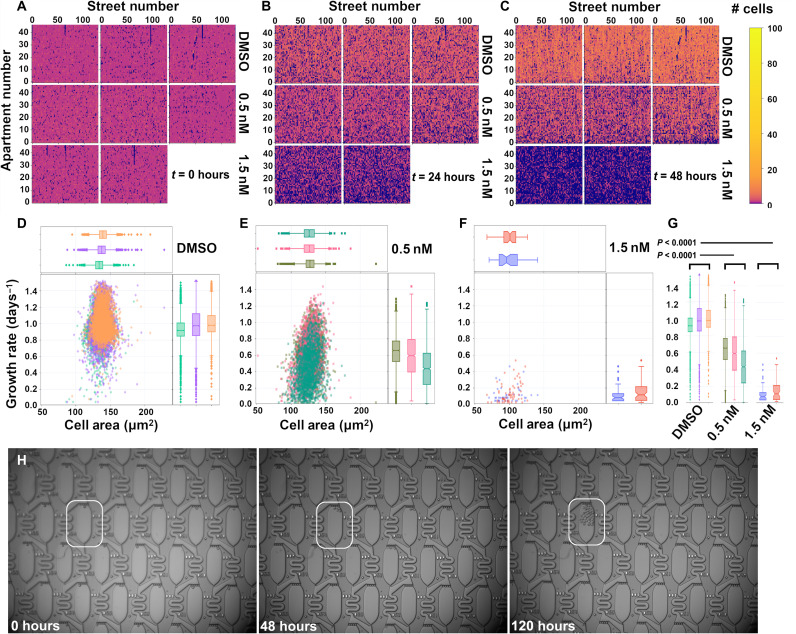
Growth rate heterogeneity due to drug response. The growth rates of MOLM-13 cells were measured in DMSO or 0.5 or 1.5 nM quizartinib over 96 hours. The cell number per apartment is plotted a heatmap at (**A**) *t* = 0 hours, (**B**) *t* = 24 hours, and (**C**) *t* = 48 hours. The heatmap colors are plotted on a log scale to better visualize the apartments with zero or one cells. The growth rate distributions are shown in several scatter plots depicting the relationship between cell division rate and mean cell area in each clone for (**D**) DMSO, (**E**) 0.5 nM, and (**F**) 1.5 nM cohorts, and significance tests for the growth rate distributions are plotted in (**G**). (**H**) Time lapse of a single drug-resistant clone emerging over 120 hours in 0.75 nM quizartinib.

During long-term culture, the flow through the chip needs to be fast enough so that the metabolic waste products from upstream apartments do not substantially affect the downstream apartments. The vacuum pressure required to achieve an optimal flow rate was found to be in the range of −30 to −70 mbar, depending on the total number of cells in the chip. When exposed to a vacuum pressure of −50 mbar, the heatmaps in Fig. 5 (A to C) reveal no apparent systematic bias in cell behavior across the chip, such as differing growth rates at positions nearer to the inlet versus the outlet. This finding supports the assumption that the growth properties of the single cells can be treated as statistically independent with regard to position inside the array. As expected, the cells thrived in the DMSO control, and a smaller fraction still grew well at the 0.5 nM conditions; however, far fewer cells survived the 1.5 nM conditions. In the 0.5 nM conditions, the median growth rates per chip were reduced to 0.55 cell divisions per day, with the middle growth rate quartiles falling in the range of 0.25 to 0.79 cell divisions per day. Unexpectedly, we still observed fast-growing cells in the drugged condition that displayed growth rates up to 1.4 cell divisions per day—these rare cells appear to be practically unaffected by the drug treatment (Fig. 5, D to G). Collectively, treatment-specific trends in growth on the chip are roughly comparable to those observed in density-optimized bulk cell culture (table S1). One notable example of a drug-resistant cell growing in the background of drug-sensitive cells is shown in Fig. 5H after several days of exposure to 0.75 nM quizartinib (see movie S1 and Fig. 5H).

We also observed a consistent, positive correlation between cell area and growth rates across the different drug conditions, likely reflecting FLT3 ITD’s established control over cell size and proliferation regulators like the mechanistic target of rapamycin (mTOR) and extracellular signal–regulated kinase (ERK) pathways, respectively (*36*). For example, the time-averaged median area per cell that was measured in the DMSO conditions was found to be 137 μm^2^, with the middle quartiles ranging from 128 to 144 μm^2^, whereas at 0.5 nM quizartinib the mean cell areas were reduced to a median of 126 μm^2^ and with the middle quartiles ranging from 119 to 133 μm^2^. However, we did not observe similar trends in the relationship between cell shape (eccentricity versus growth rate) as shown in fig. S2. These relationships are further exemplified in fig. S3, which shows a parallel coordinate plot linking the individual cell trajectories to the size dependence. The similarity of the growth trajectories across different cohorts was also classified with *t*-SNE (*t*-distributed stochastic neighbor embedding) plots in fig. S4.

## DISCUSSION

We have developed a high-throughput live-cell biology platform that can establish and maintain highly reproducible cellular architectures on chip for multiple days. This platform enables the analysis of phenotypic heterogeneity at the necessary scales for measuring low-frequency variants in a population, such as cells that are resistant to a drug or have other rare morphological features, and complements other methods for quantifying short-term single-cell drug responses and its relationship to transcriptional programs (*38*).

There are potential areas for improving this platform, such as by functionalizing the substrates with adhesive versus nonadhesive patches at selective positions in the device and by using frit structures based on porous hydrogels (*39*), which can help support and better constrain adherent and suspension cell cultures. We cannot completely discount the possibility that the drug exposure may depend on the number of cells in each apartment, causing some cell density–dependent variation in drug responses; however, we do not expect this variability to be any more notable than in traditional bulk cultures, because the flow speed is sufficient to replace the entire chip volume with fresh media once every minute, and the drug consumption rate is likely much lower. A current limitation of this platform is the inability to retrieve live cells; however, it is possible to use reversible lids (*40*–*41*) that can be peeled off at a desired endpoint to enable access to the sample with a robotic clone picker (*41*). Our current approach uses chips fabricated in silicon/glass; however, because the microfluidic pattern is just a single layer, it is possible to fabricate similar chips in polydimethylsiloxane and plastic. We aimed to showcase a typical experiment that can be conducted by one user within 1 week, and thus, we only present one complete dataset performed at the same time, shown in Fig. 5. One of the chips cracked midway through that experiment, which prevented us from obtaining a full time series for the 1.5 nM cohort in triplicate. Although we observed similar behavior at this drug condition in different experiments, in Fig. 5, we chose to include only the measurements from a single experiment to highlight the statistical power and capabilities that can be achieved with this platform. Although not reported here, we also expect that, in the future, these high-throughput phenotyping capabilities can be combined with the selective patterning of DNA primers inside the apartments to enable highly parallel transcriptome measurements alongside image-based phenotyping for potential applications in single-cell functional genomics assays.

## MATERIALS AND METHODS

### Experimental design

#### Chip fabrication

Microfluidic chips are fabricated on 6-inch wafers using DRIE to form the channel walls, as previously described (*41*, *42*). Photoresist (Shipley 1813) is spun onto the wafers at 500 rpm for 5 s and 4000 rpm for 60 s, baked at 115°C for 60 s, exposed to 80 to 100 mJ/cm^2^ in a Karl Suss MA6 mask aligner, and then developed in Microposit MF-319 developer for 30 s. The wafers are then thoroughly cleaned and etched to a depth of 15 to 20 μm in the DRIE (SPTS Pegasus Deep Silicon Etcher). The photoresist mask is then stripped and cleaned in piranha solution (3:1 H_2_SO_4_ to H_2_O_2_ at 200°C). Next, a 15-μm-thick layer of AZ 9260 photoresist is spun onto the backside of the wafer at 500 rpm for 5 s and 1800 rpm for 60 s, baked at 110°C for 60 s, exposed to 4000 mJ/cm^2^, and developed for 300 s in AZ 400K 1:4 developer. This layer is used to create through-silicon vias to establish the inlets and outlets and dice the chips. The photoresist is then stripped and thoroughly cleaned as described previously. Last, we anodically bond borosilicate glass to the silicon microchannels at 300°C for 3 hours. In total, each wafer yields 12 devices (chips), which have dimensions of 30 mm × 25 mm.

#### Microfluidic setup

Custom-made chip holders were machined in Aluminum (Protolabs, MN), comprising a bottom holder and a top-viewing window. The bottom piece contained ^1^/_4_″-28 threaded holes to allow for connection to be made to the chips with screw-in Luer locks (BSFTLL-6005, Nordson Medical). The chip holders were also anodized (Surtronics, Raleigh, NC) to ensure that they would last inside the high humidity environment of a cell culture incubator for long durations. The chip holders were placed onto a custom stage adapter and mounted on an ASI-RAMM microscope (Applied Scientific Instrumentation, Eugene, OR), which contains an automated focus drive, an objective changer, and a filter changer. Fluid was introduced to the chip with an Elvesys pressure controller (OB1 MK3+, Paris, France) that applied vacuum pressure at the outlet.

#### Cell culture

MOLM-13 AML cells (*43*) were obtained from the Wood laboratory. Cells were maintained in RPMI 1640 medium (Gibco 11875-093) supplemented with 10% fetal bovine serum (Gibco 10347-028) and penicillin/streptomycin (Gibco 15140-122) in a 5% CO_2_ environment. Cells were passaged in T25 flasks and centrifuged for 5 min at 350 relative centrifugal force before subculturing to maintain a density range of 2.0 × 10^6^ to 3.0 × 10^6^ cells per milliliter. A new thaw of cells was used every 8 weeks to minimize genetic drift. Counting and viability with 0.4% trypan blue were determined with a Countess II instrument (Thermo Fisher Scientific). Quizartinib (AC220) was obtained from Selleck Chemicals LLC.

#### Cell loading

Cells were loaded onto the chip by pipetting a 20-ml aliquot into screw-in Luer locks positioned on the inlet side, after which the cells were infused into the chip by applying 20- to 30-mbar vacuum pressure to the outlet side using a syringe body that was attached to a rubber stopper. The microfluidic architecture consists of one inlet and one outlet, which feed into the active area of the chip by successive flow division in a binary tree, leading to 128 parallel streets with 47 apartments in series. The loading time typically required 3 to 5 min for the cells to reach the last row of apartments in each street, corresponding to a loading rate of about 20 cells per second. After the cells were trapped in each constriction, the Luer locks on the inlet side were rinsed at least three times by replacing the fluid with fresh cell culture media. To eliminate any remaining cells that were stuck in the Luer lock or on the chip surface, we irradiated the Luer locks with ultraviolet C using a 270-nm light-emitting diode attached to a heat sink (Irtronix, Torrance, CA)—this provided a lethal radiation dose to any nonspecifically adhered cells and prevented the chips from being invaded with cells at later time points. Last, the cells were squeezed through the constrictions by applying a brief (~1-s) pressure pulse in the range of 300 to 800 mbar to the outlet, similar to previously reported techniques (*40*). The chips were then disconnected from the imager and put into the incubator.

#### High-throughput microscopy

We developed custom python codes to rapidly take images of each apartment. The algorithm involved first identifying three crosshairs on the chip to establish the equation of a plane, next creating a stage position list containing the *XY* position and optimal focal plane for each image, then taking images of each apartment, and finally saving and naming the images in custom formats to render them compatible with the computer vision algorithms. The software used to image the chips is provided at GitHub (*34*), and because they are based on a Python wrapper for Micro-Manager (*44*), the program is easily adapted for most standard robotic microscopes.

#### Fluorescence imaging

Chips were loaded with MOLM-13 cells and cultured in 0.2 μm–filtered R10 media at 37°C and 5% CO_2_. At 72 hours, 5 μl of Hoechst 33258 (0.1 mg/ml) was added to ~50 μl of media at the microfluidic inlet port and flowed onto the chip using negative pressure (−100 mbar) applied at the microfluidic outlet. Constant flow was maintained for 10 to 15 min to stain cell nuclei, followed by rinsing with media. Similarly, 5 μl of PE-conjugated anti-CD45 (0.2 mg/ml; Invitrogen) was flowed in, incubated, and rinsed before imaging. Multichannel images were collected in brightfield and using standard 4′,6-diamidino-2-phenylindole (DAPI) and Texas Red filter sets.

### Statistical analysis

#### Image analysis

We developed custom python codes to rapidly analyze the images and extract cellular phenotypic properties in a computationally efficient manner. Our cell extraction algorithms make use of the Mask R-CNN image segmentation model (*45*), which is designed to identify objects in images without the need for pixel classification postprocessing. This is an advance on previous methods for biological image segmentation (*46*–*47*) that enabled us to compose a simple pipeline for cell quantification using a minimal amount of training data. In a separate study, we quantified the superior performance of Mask R-CNN segmentation relative to supervised segmentation algorithms and statistical methods (*48*). Similarly, the SVHN (street view housing number) (*49*) model is an architecture for digit classification that we used to determine the apartment identifiers etched into the chips.

Our pipeline consists of three separately applied models, where the first is used to identify a key point within each apartment image (hereto referred to as a “marker”), given an image containing multiple apartments (i.e., raw microscope images). Images of individual apartments were then extracted using these markers. Because the raw microscope images often have slight rotations, the relative positions of the identified markers in adjacent apartments were used to infer an overall rotation of the images to be inverted before further decomposing the individual apartment images. The apartment images were then registered against a template image to remove small translations. The digit identifiers for each apartment, with no rotations or translations, were extracted on the basis of fixed offsets from the marker position. Fixed offsets are determined relative to several chip landmarks and need to be updated whenever the chip form factor is altered. Identification of individual cell objects is performed on the basis of the entire apartment image, but segmented results are filtered to the apartment and trap areas, again using fixed offsets from the marker, as a way to prohibit erroneous classification of debris within microfluidic channels.

Training for the cell segmentation model included 814 annotated images, and the Mask R-CNN model trained was initialized to a weight set resulting from pretraining over the COCO (*50*) image dataset, a feature provided by the Matterport implementation (*33*). Training also included an augmentation pipeline consisting of image flips, affine rotations, random croppings, contrast transform, and blurring. The marker identification model was trained in a very similar fashion but required only 70 annotated images because the associated classification task was simpler. By contrast, the digit recognition model required far more training data (9375 annotated images), although this annotation task was much less time consuming because the individual digit images only needed to be assigned a class; hence, bounding boxes or object masks were not required.

We have also developed a dashboard visualization tool that allows the growth rates and other properties to be viewed at the experiment level, individual apartment level, and array levels. More details on the software package can be found at GitHub (*30*).

#### Data analysis

The data presented in Figs. 3 and 5 are limited to apartments starting from a single cell and having at least one cell in the apartment at each time point. This led to substantially fewer data points for the 1.5 nM quizartinib cohort, where a majority of cells did not survive the drug treatment over several days. The growth rates are determined by fitting the raw trajectories to an exponential with base 2, i.e., *p*(*t*) = *p*_0_2^λ*t*^, where λ is the cell division rate and *t* is the time measured in days. This simple exponential model ignored any hysteretic effects and therefore should be considered as an approximation of a given clone that maintains positive continuous growth throughout the experiment. The calculated growth rates are likely to be a lower bound, because the image segmentation models begin to miss cells in apartments that are very crowded, as shown in Fig. 3B. The *P* values shown in Fig. 5G are calculated by random sampling of the growth rates of 1000 clones in each cohort and indicate that the growth distribution of the drugged conditions is statistically significant compared to the DMSO controls.

## Supplementary Materials

### This PDF file includes:

Supplementary Theory Figs. S1 to S4 Table S1 Legend for movie S1

## Notes

View/request a protocol for this paper from *Bio-protocol*.
