# Supplementary material for: Massively parallel quantification of phenotypic heterogeneity in single-cell drug responses
Source: Sci Adv. 2021 Sep 17;7(38):eabf9840. doi: 10.1126/sciadv.abf9840 (PMC8448449; doi:10.1126/sciadv.abf9840)
Supplement: Supplementary file 1 — Supplementary Theory Figs. S1 to S4 Table S1 Legend for movie S1 [file sciadv.abf9840_sm.pdf]

Science Advances
AAAS

Supplementary Materials for

Massively parallel quantification of phenotypic heterogeneity in single-cell drug responses

Benjamin B. Yellen*, Jon S. Zawistowski, Eric A. Czech, Caleb I. Sanford, Elliott D. SoRelle, Micah A. Luftig, Zachary G. Forbes, Kris C. Wood*, Jeff Hammerbacher*

*Corresponding author. Email: yellen@duke.edu (B.B.Y.); kris.wood@duke.edu (K.C.W.); hammer@hammerlab.org (J.H.)

Published 17 September 2021, *Sci. Adv.* **7**, eabf9840 (2021)
DOI: 10.1126/sciadv.abf9840

The PDF file includes:

Supplementary Theory
Figs. S1 to S4
Table S1
Legend for movie S1

Other Supplementary Material for this manuscript includes the following:

Movie S1

Supplementary Theory

Laminar flow hydrodynamic networks can be modeled like electrical circuits, where the pressure, flow rate, and hydrodynamic resistances are analogous to voltage, current, and electrical resistances. Ladder (or mesh) networks are comprised of two types of resistors, including those aligned parallel to the main flow path, i.e., R_A and R_S, (the rails of the ladder) and those aligned perpendicular to the main flow path, i.e., R_B (the rungs of the ladder). The flow distribution can be solved by setting up continuity equations at each branch point in the array. From there, we apply a constant pressure drop, ΔP, parallel to the flow direction across each array period. This system of equations thus reduces to solving the pressure at 4 nodes in the minimum unit cell, which are given by:

$$\begin{bmatrix} R_B^{-1}+R_A^{-1}+R_S^{-1} & -R_B^{-1} & -R_A^{-1}-R_S^{-1} & 0 \\ -R_B^{-1} & R_B^{-1}+R_A^{-1}+R_S^{-1} & 0 & -R_A^{-1}-R_S^{-1} \\ -R_A^{-1}-R_S^{-1} & 0 & R_B^{-1}+R_A^{-1}+R_S^{-1} & -R_B^{-1} \\ 0 & -R_A^{-1}-R_S^{-1} & -R_B^{-1} & R_B^{-1}+R_A^{-1}+R_S^{-1} \end{bmatrix} \begin{bmatrix} P_{i,0} \\ P_{i+1,0} \\ P_{i,1} \\ P_{i+1,1} \end{bmatrix} = \Delta P \begin{bmatrix} R_A^{-1} \\ R_S^{-1} \\ -R_A^{-1} \\ -R_S^{-1} \end{bmatrix}$$

where $P_{i,0}$, $P_{i,1}$, $P_{i+1,0}$, and $P_{i+1,1}$ are the four unique nodes of the unit lattice.

The pressures at each node can be solved by inverting Eq. (1) to yield a generic solution in terms of the pressure at an arbitrary point, in this case chosen as $P_{i,0}$:

$$P_{i,0} = P_{i,0}$$

$$P_{i+1,0} = P_{i,0} - \frac{1}{2}\frac{R_A^{-1} - R_S^{-1}}{R_A^{-1} + R_B^{-1} + R_S^{-1}}\Delta P$$

$$P_{i,1} = P_{i,0} - \frac{1}{2}\frac{R_B^{-1} + R_S^{-1}}{R_A^{-1} + R_B^{-1} + R_S^{-1}}\Delta P$$

$$P_{i+1,1} = P_{i,0} - \frac{1}{2}\Delta P$$

We can then determine the ratio of flow along the two lateral paths, Q_B, relative to the flow through the apartment, Q_A, which are given by:

$$\frac{Q_A}{Q_B} = \frac{R_B + R_A}{R_S - R_A}$$

Since this ratio changes sign as a function of the relative magnitude of R_A and R_S, this result indicates that there are two regimes of fluid flow. When $R_S > R_A$, which is the typical scenario for previously studied trapping designs, the flow ratio is positive and approaches a singularity when R_S is nearly equal to R_A. This singularity defines a critical point where there is zero flow through the lateral branches, R_B, and all of the flow moves solely through the R_A and R_S paths, practically in straight lines. An alternative way to think of this phenomenon is that the pressure at the adjacent nodes $P_{i,0}$ and $P_{i,1}$ are equal when R_A and R_S have equal resistance, leading to zero flow in the lateral branches.

The other flow regime, which has not previously been reported, occurs when $R_S < R_A$, which leads to the ratio in Eq. (3) becoming negative. The significance of this sign inversion is that the flow through the lateral branches, Q_B, is assigned in the wrong direction. In this flow regime, all of the fluid joins together at the branch point and flows through the trap, which is a perfect trap from a mathematical sense.

Supplementary Figures

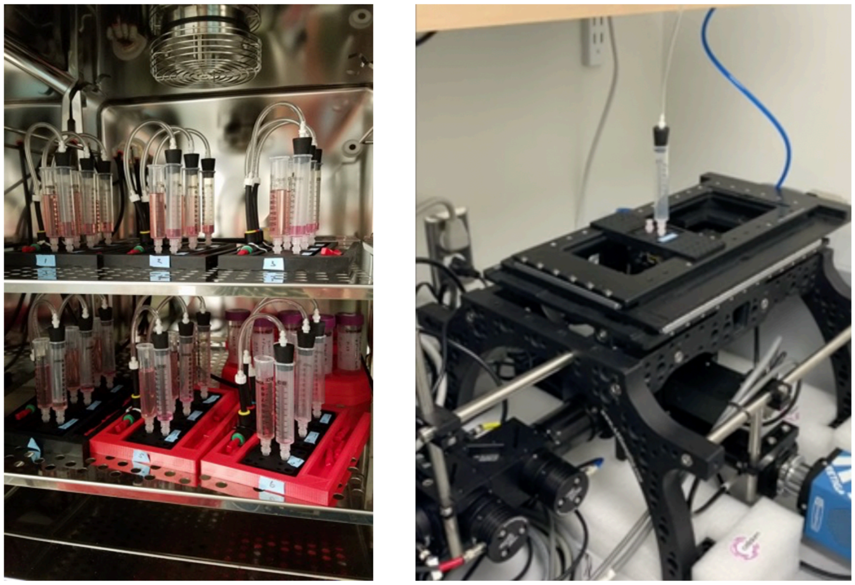

(a) (b)

Figure S1. The incubator system used for maintaining chips with media is shown (a). Continuous media flow through the chips was achieved by pulling vacuum at the outlet, while allowing the media at the inlet to be exposed to the gas- and temperature-controlled environment of the incubator. External to the environment, the pressure was maintained by 6 vacuum regulators, which were divided with 3-way splitter manifolds, enabling up to 18 chips to be contained simultaneously. The chip on the imager is shown (b).

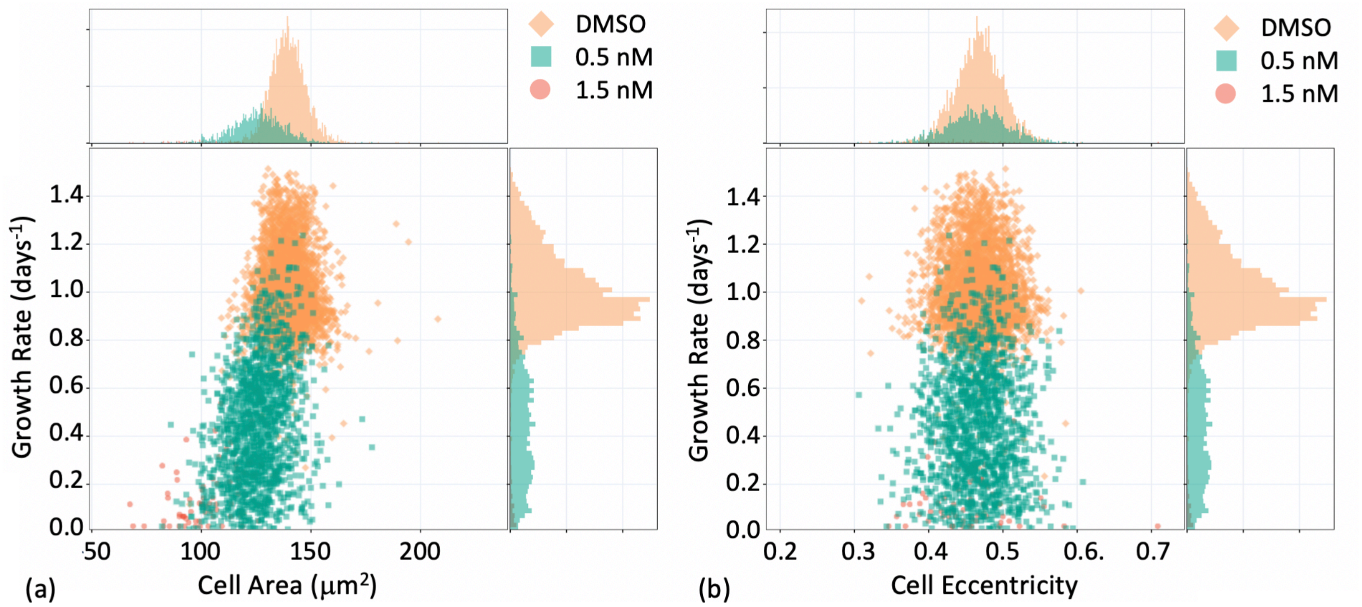

Figure S2. Relationships between cell size (a) and shape (b) versus clonal growth rate is shown for an example chip in the 1.5 nM, 0.5 nM, and DMSO cohort. There is a positive correlation between cell size and growth rate, however a correlation between cell shape and growth rate was not observed.

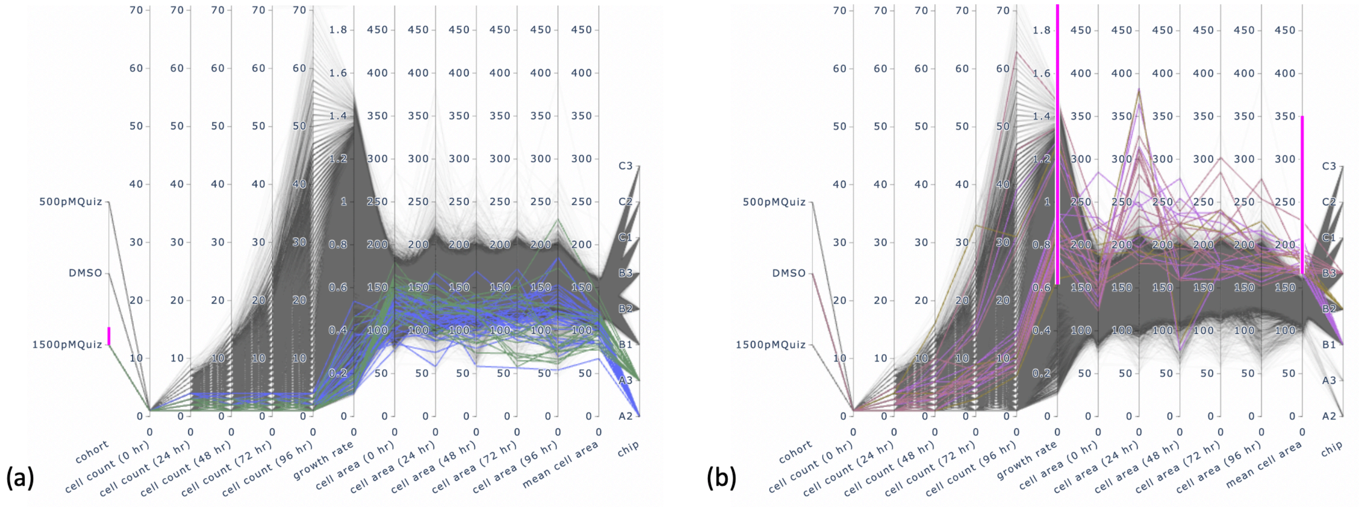

Figure S3. The parallel coordinates plot shows the growth and size trajectory for each cell identified in the different cohorts and chips and is used as compact method for visualizing linked phenotypic attributes. The pink bars represent the filtering to highlight the linked attributes. (a) The growth trajectories of the 1.5 nM quizartinib cohort, which are highlighted in the blue and green trajectories, show significantly lower growth rates and smaller time-averaged cell size. (b) A subset of cells are filtered to display the cell trajectories with growth rates greater than 0.66 cell divisions per day and with cell areas greater than 150 μm^2.

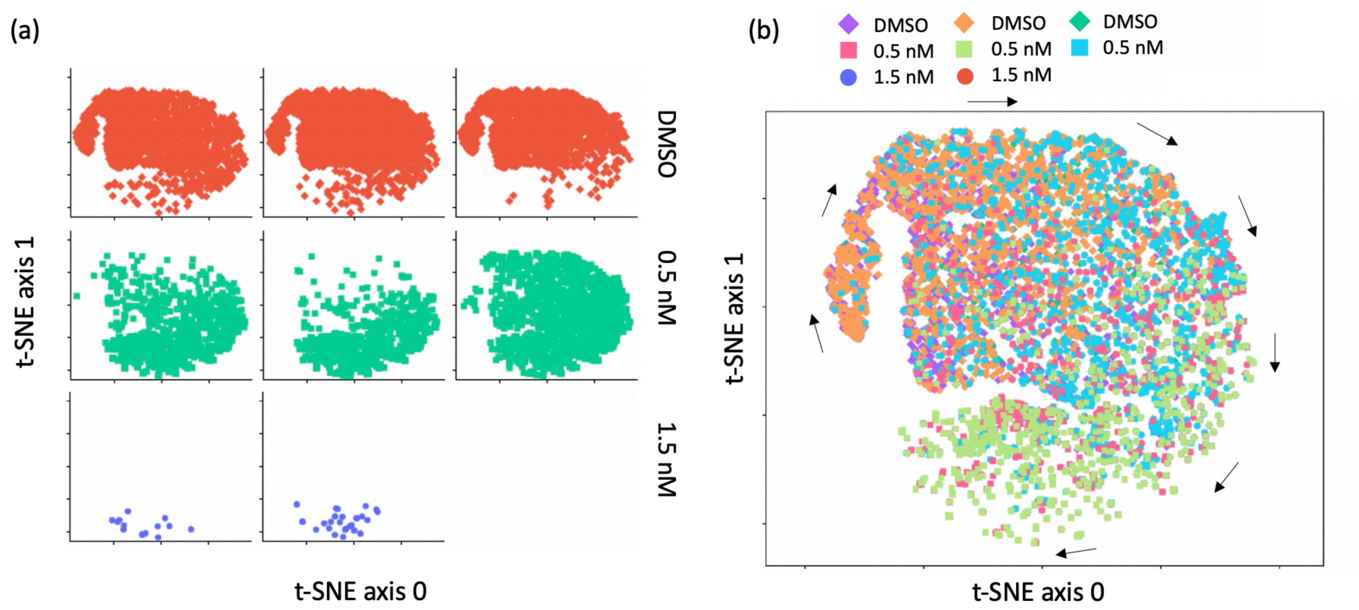

Figure S4. t-SNE plot is shown for the cell trajectory data. The arrows denote the direction of decreasing cell division rate. The individual cluster map for each chip is shown in (a) and the combined data overlaid is shown in (b).

Bulk Culture	DMSO	0.5 nM Quizartinib	1.5 nM Quizartinib
**Cell division rate**	1.25 +/- 0.17	0.73 +/- 0.05	0.56 +/- 0.06

Table S1. Treatment group growth rates in bulk culture. The population cell division rate was measured for MOLM13 cells subjected to quizartinib treatment in bulk cell culture over 72 hours in 6-well plates at a starting density of 0.88 +/- 0.023 x 10^6 cells per mL. All data are presented as mean +/- standard deviation measurements from n=3 replicates per condition. All measurements were performed by counting cells using standard hemocytometers with Trypan Blue staining to assess viability. The population averaged cell division rate of MOLM-13 cells display remarkable dependence on starting density and drops off significantly at lower or higher starting densities.

Supplementary Movies

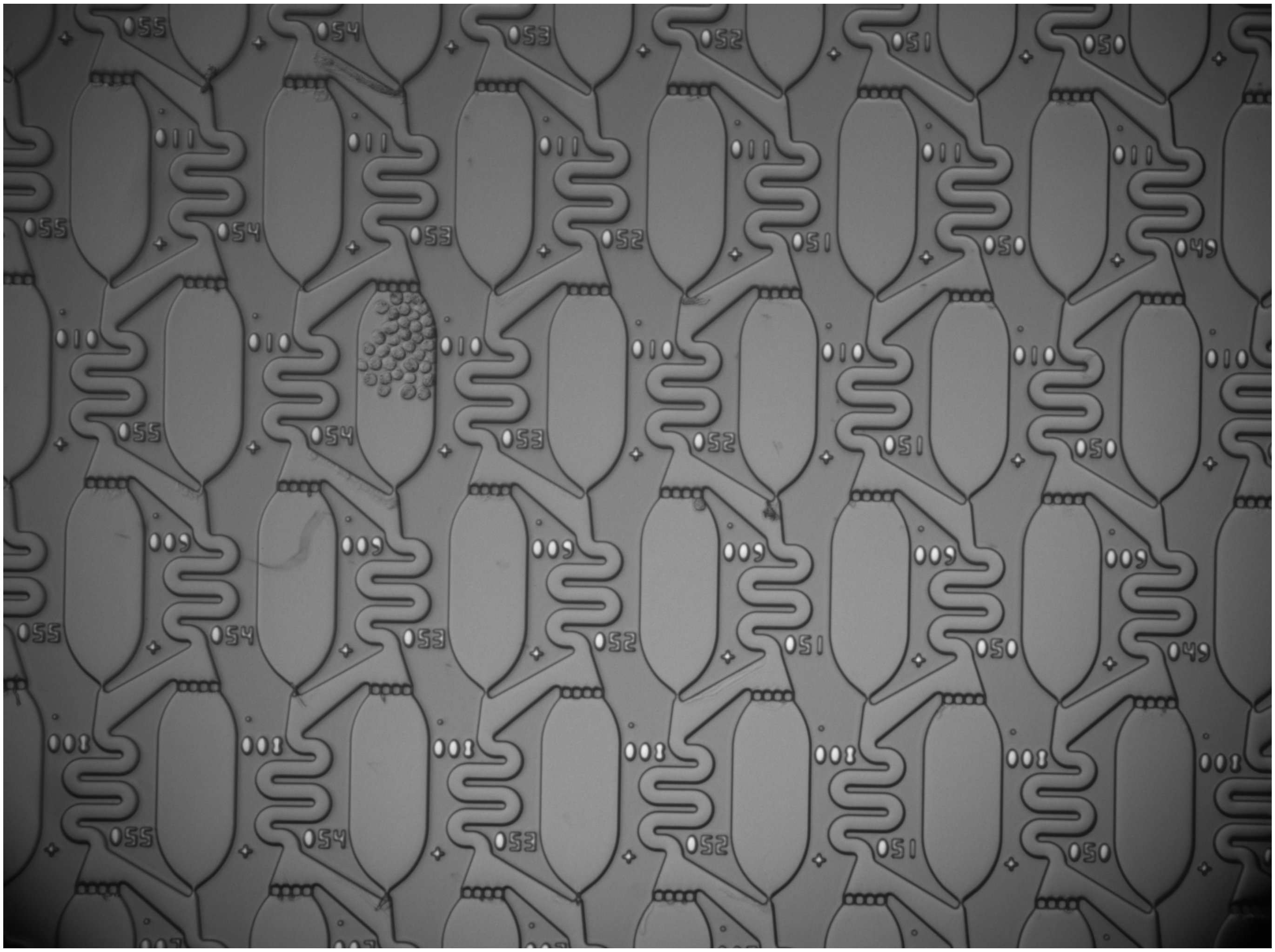

Movie S1. This time-lapse video shows a single drug-resistant MOLM-13 clone emerging after 120 hr of continuous exposure to 0.75 nM quizartinib.
